# Comparability Evaluation of Three Benchtop Glucose Analyzers With the Recently Withdrawn YSI 2300 Stat Plus

**DOI:** 10.1177/19322968241230337

**Published:** 2024-02-08

**Authors:** Gareth J. Dunseath, Iulius-Dumitru Vatavu, Stephen D. Luzio

**Affiliations:** 1 Diabetes Research Group, Faculty of Medicine, Health and Life Science (FMHLS), Swansea University, Swansea, UK

**Keywords:** analyzer, glucose, comparison

## Abstract

**Background:**

We compared the performance of three currently available laboratory benchtop glucose analyzers with the outgoing YSI 2300 Stat Plus.

**Methods:**

Plasma samples (100), across a wide glucose concentration range were analysed on the YSI 2500, Randox daytona+ (glucose oxidase) and EKF Biosen in a single laboratory and compared to the YSI 2300 Stat Plus.

**Results:**

All three analyzers showed good agreement with the YSI 2300 Stat Plus, and only a small bias (≤1% YSI 2500 and Randox daytona+, 4.6% EKF Biosen) was observed for each analyzer. None of the three comparator analyzers were affected by either proportional or constant bias, thus no significant differences between the YSI 2300 Stat Plus and the comparator methods were identified.

**Conclusions:**

The results from this study suggest all could be considered as suitable reference laboratory glucose analyzers and replacements for the recently withdrawn YSI 2300 Stat Plus.

## Introduction

In July 2021, YSI withdrew support for the YSI 2300 Stat Plus glucose and lactate analyzer, with parts no longer available after this date, with the manufacturer recommended replacement being the 2900D Biochemistry analyzer.^1^ A white paper report published by YSI in 2017 showed that the 2900D displayed analytical comparability with the 2300 Stat Plus across a wide range of glucose concentrations.^2^ However, the 2900D is not currently Food and Drug Administration (FDA) approved, and it provides a multi-chemistry format and is thus a more complex analyzer than the 2300 Stat Plus. YSI also market the YSI2500 which is a limited version of the 2900D with some of the functionality removed, allowing measurement of glucose and lactate only. The YSI 2500 Glucose/Lactate analyzer is the cost-effective alternative to the 2900D analyzer, supporting identical analytical methodologies for glucose and lactate assays only.

For many years, the YSI 2300 Stat Plus was the instrument of choice used for most blood glucose meter (BGM) accuracy validation studies and for lot calibration of glucose test strips. The discussion over which glucose analyzer should replace the YSI 2300 Stat Plus as the “Gold standard” method has been ongoing, with no formal consensus reached as yet.^3-5^

A variety of alternative handheld devices and benchtop lab analyzers, offering glucose measurement only (eg, HemoCue Glucose 201+. Arkray Glucose Analyzer 1172) and glucose measurement as part of a multi-chemistry format (eg, ABL90 Flex Radiometer, Siemens EPOC, GEM Premier 5000, Abbott i-STAT, Roche Integra Cobas 400 plus, Super GL Compact), are in use and have been summarized previously.^4^ A number of these analyzers currently have FDA clearance.

The choice of reference analyzer for BGM accuracy validation (as well as for diagnostic and metabolic challenge testing) should consider the importance of the accuracy of the reference laboratory analyzer, as has previously been described^6^; however ease of use, speed of results generation, analyzer footprint, sample type, and volume are among other important factors that should also be considered.

Here, we describe the findings of a comparison of the performance of three currently available laboratory benchtop glucose methods: the YSI 2500 glucose lactate analyzer, the Randox daytona+, and the EKF Biosen glucose lactate analyzer, with the YSI 2300 Stat Plus. All of the abovementioned analyzers were selected due to their common glucose oxidase measurement technology and their availability for testing in the Diabetes Research Group Laboratory (Swansea University, UK) during the study period.

## Methods

This evaluation study was independently reviewed and approved as non-research (Quality Assurance/Evaluation) by Swansea University and Swansea Bay University Health Board Joint Study Review Committee.

A total of 100 plasma samples, spanning a wide concentration range, were collected in fluoride oxalate blood collection tubes. All plasma samples were stored at −70°C following collection, before being analyzed in batches. Each batch of samples was analyzed on all four analyzers on the same day.

### Analyzers

#### YSI 2300 Stat Plus

The YSI 2300 Stat Plus (YSI Incorporated, Yellow Springs, Ohio) provides a rapid measurement of glucose using a glucose oxidase/l-Lactate enzyme hydrogen peroxide sensor to determine glucose levels in 25 µL of sample.

The YSI 2300 Stat Plus was used as the reference comparator method in this study.

#### YSI 2500

The YSI 2500 (YSI Incorporated) offers manual or automated sample handling to measure glucose in blood or plasma samples using 25 µL of sample.^7^ The analyzer uses the same enzyme measurement technology as the 2300 Stat Plus.

#### Randox daytona+ (Glucose oxidase)

The Randox daytona+ (Randox Laboratories, Crumlin, UK) is a fully automated clinical chemistry analyzer.^8^ Glucose was measured using a glucose oxidase method (GL83198) utilizing direct photometry. The assay method uses less than 10 µL of plasma or serum.

#### EKF Biosen

The EKF Biosen (EKF Diagnostics, Penarth, UK) is an automated benchtop analyzer, utilizing enzymatic-amperometric chip technology, with glucose oxidase immobilized onto the chip.^9^ A 20 µL sample of blood, plasma, or serum is collected into a 20 µL capillary tube and transferred to a sample cup pre-filled with hemolysis solution for analysis.

### Statistical Analysis

Agreement with the 2300 Stat Plus was established by:

1. Scatterplot (with *R*^2^),
2. Bland Altman plot (absolute and percentage mean difference),^10^
3. Passing and Bablok analysis (proportional and constant bias).^11^

## Results

Mean (range) glucose concentration was 214.0 mg/dL (54.9-460.8 mg/dL) when measured using the YSI 2300 Stat Plus. Corresponding mean (range) glucose concentrations when measured on the YSI 2500, Randox daytona+, and EKF Biosen were 213.9 mg/dL (65.5-466.2 mg/dL), 210.9 mg/dL (66.6-483.5 mg/dL), and 220.4 mg/dL (67.9-449.8 mg/dL), respectively.

**Figure 1.**
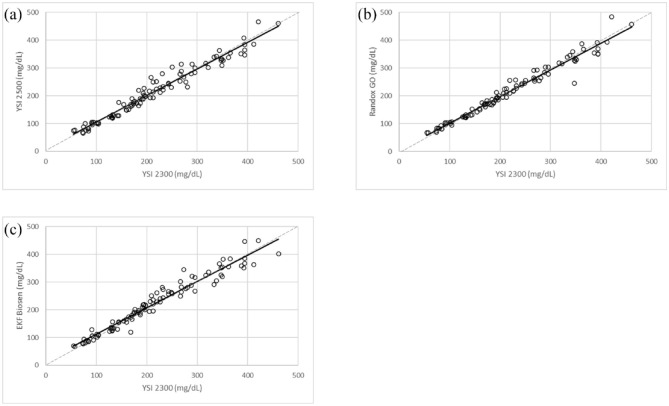
Scatterplots of the comparator glucose analyzers with the YSI 2300 Stat Plus: (a) YSI 2500, (b) Randox daytona+, and (c) EKF Biosen. Abbreviation: GO, glucose oxidase.

All three analyzers displayed a very high *R*^2^ value >0.95 compared with the YSI 2300 Stat Plus (as shown in scatterplots, Figure 1).

**Figure 2.**
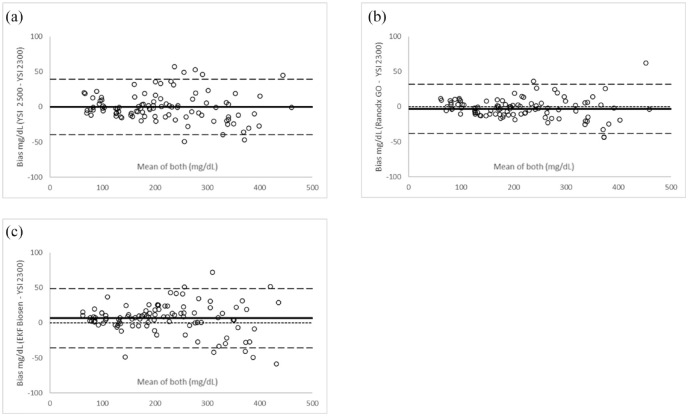
Bland Altman plots of the comparator glucose analyzers with the YSI 2300 Stat Plus: (a) YSI 2500, (b) Randox daytona+, and (c) EKF Biosen.

Compared with the YSI 2300 Stat Plus, all three comparators had a small mean difference of −0.13, −3.12, and 6.41 mg/dL; YSI 2500, Randox dayona+, and Biosen, respectively, or <1.0% for YSI 2500 and Randox daytona+ and 4.6% for Biosen across the entire concentration range (see Table 1 and Figure 2).

**Table 1.** Summary of *R*^2^ and Mean Difference for the Comparator Analyzers With the YSI 2300 Stat Plus.

	*R* ^2^	Mean difference (mg/dL)	Mean difference (%)
2500	0.959	−0.13	0.98
daytona+	0.9675	−3.12	−0.67
Biosen	0.9527	6.41	4.60

Passing and Bablok analysis demonstrated that none of the three comparators were affected by either proportional or constant bias (see Table 2), thus no significant differences between the YSI 2300 Stat Plus and the comparator methods were identified.

**Table 2.** Passing and Bablok Analysis Summary for the Comparator Analyzers With the YSI 2300 Stat Plus.

	Intercept		Slope	
	Estimate	Lower/upper	Estimate	Lower/upper
2500	2.044	−4.598/10.59	0.9716	0.932/1.019
daytona+	3.472	−3.886/8.224	0.9693	0.9361/1.008
Biosen	7.217	−6.263/14.91	1.003	0.9567/1.053

## Discussion

The YSI 2300 Stat Plus has long been recognized as the “Gold standard” reference glucose analyzer for BGM accuracy validation studies; however, it has now been withdrawn from service. As such, agreement on whether selecting a single replacement reference glucose analyzer is required.

In this study, we have compared the performance of three currently available laboratory benchtop analyzers with the outgoing YSI 2300 Stat Plus across a wide glucose concentration range in a single laboratory.

All three analyzers showed good agreement with the YSI 2300 Stat Plus, across the glucose range tested. Only a small bias was observed for each analyzer (<1% for YSI 2500 and Randox daytona+, 4.6% for Biosen). The analyzer with closest agreement in concentration was the YSI 2500; however, this is to be expected given their shared measurement technology.

Both the YSI 2500 and the Biosen were easy to use and generated quick results; however, due to the Randox daytona+ being a multi-chemistry analyzer, the procedure was slower and requires more user training.

Strengths of this work include using a large number of samples across a wide concentration range and the work being performed in a single laboratory. The study does however have a number of limitations, largely due to the study design being pragmatic in nature rather than following strictly to the standards of either ISO 15197 or CLSI POCT12-A3. As such, the study findings could benefit from including analysis of whole blood samples (where appropriate for the assay method), extending the glucose concentrations further into the hypoglycemia range by manipulating whole blood samples collected without a glycolytic inhibitor. In addition, inclusion of a certified reference material would strengthen the data. The findings would also benefit from being confirmed by performing a similar comparison in other laboratories.

## Conclusions

The YSI 2500, Randox daytona+, and the EKF Biosen all demonstrated very similar performance to the recently withdrawn YSI 2300 Stat Plus. The results from this study suggest that all show potential to be considered for further testing as suitable reference laboratory glucose analyzers.
